# Mussel‐Inspired Adhesive Polydopamine‐Functionalized Hyaluronic Acid Hydrogel with Potential Bacterial Inhibition

**DOI:** 10.1002/gch2.201900068

**Published:** 2019-11-18

**Authors:** Qi‐Hang Yu, Chen‐Ming Zhang, Zhi‐Wei Jiang, Si‐Yong Qin, Ai‐Qing Zhang

**Affiliations:** ^1^ School of Chemistry and Materials Science South Central University for Nationalities Wuhan 430074 China

**Keywords:** adhesives, bacterial inhibition, free radical scavenging, hydrogels, polydopamine

## Abstract

Hyaluronic acid (HA)‐based hydrogels have been receiving increasing attention for wound management. However, pure HA hydrogels usually exhibit weak mechanical strength and poor anti‐infection. Herein, a hybrid HA‐based hydrogel (PDA‐HA) comprised of polydopamine (PDA) and thiolated hyaluronic acid (HA‐SH) is developed based on the Michael addition reaction. The introduction of PDA into HA hydrogel can decrease the critical gel concentration, improve the cell affinity and tissue adhesion, as well as endow the hydrogel with efficient free‐radical scavenging ability. Combining the merits of good biocompatibility and moist environment from HA hydrogel with excellent tissue adhesiveness and free radical scavenging capability from PDA, this cross‐linked PDA‐HA hybrid hydrogel exhibits great potential for creating antimicrobial wound medical dressings.

## Graphical abstract

A multifunctional hydrogel (PDA‐HA) comprised of polydopamine (PDA) and thiolated hyaluronic acid (HA) is developed via Michael addition reaction. The obtained hybrid hydrogel exhibits good tissue adhesion, effective free radical scavenging, and potential bacterial inhibition, which may exhibit great potential for application as a wound dressing.

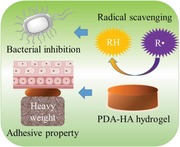

Originated from the biomimetic nature, excellent biocompatibility and permeable structure, water‐rich hydrogels from natural polymers have been extensively expanded in biomedical applications such as drug delivery, biosensor, 3D cell culture, regenerative medicine and tissue engineering matrices. For instance, pH‐sensitive alginate hydrogel has been developed to load vanillin and achieved the pulsatile drug release.^1^ Thermosensitive chitosan‐based hydrogel could encapsulate exogenous recombinant human stromal cell‐derived factor‐1 alpha to recruit mesenchymal stem cells for corneal epithelium regeneration.^2^ Carrageenan based hydrogel has been exploited in the fields of bone and cartilage tissue engineering.^3^ In recent years, natural polymer hydrogels have been demonstrated with the potential as wound dressings because they can afford a moist environment to promote the wound healing.^4^, ^5^, ^6^, ^7^, ^8^ Especially, these hydrogel‐based dressings present various properties such as absorbing exudates from wounds, in situ encapsulating drugs and filling irregular wound sites, etc. Among them, hyaluronic acid (HA)‐derived hydrogel is one of the ideal candidates due to its inherent biological homology, feasible structure modification, as well as good degradability.^9^, ^10^ The high water retention capacity and high viscoelasticity of HA also allow it to be suitable for wound dressings.

To construct desirable HA‐based hydrogels, different strategies have been established. Relying on the intermolecular noncovalent interactions, the physical cross‐linking HA hydrogels emerged. Rodell et al. developed a shear‐thinning and self‐healing HA hydrogel due to the host‐guest interaction between β‐cyclodextrin (β‐CD) and adamantine (AD) appended HA.^11^ The hydrogel properties could be rationally tailored through the material concentration, degree of modification, and the ratio of β‐CD to AD. Nevertheless, the physical hydrogels may exhibit undesirable mechanical properties and suffer from premature dissociation. Chemical cross‐linking of pendant reactive groups by coordination chemistry or polymerization ignites an alternative route to fabricate HA‐based hydrogels. In the meanwhile, transition metal ions or exogenous cross‐linking agents usually involve,^12^ which may impair the systemic biocompatibility due to their possible cytotoxicity. Recently, various compatible strategies have been proposed to develop biocompatible hydrogels, such as enzyme‐mediated covalent linking,^13^, ^14^ disulfide cross‐linking,^15^, ^16^ and Diels–Alder reaction.^17^, ^18^ Indeed, these chemical cross‐linked hydrogels exhibit good mechanical property and are cytocompatible. However, their further applications are restricted by their limited functions. Thus, endowing HA hydrogels with multiple functions seems to be very promising, and fabricating HA‐based functional hydrogels with good biocompatibility is still challengeable.

In this study, a multifunctional hydrogel (PDA‐HA) comprising of polydopamine (PDA) and thiolated hyaluronic acid (HA‐SH) is developed (**Scheme** 1). PDA was introduced because of its good biocompatibility, strong tissue adhesion and effective free radical scavenging ability. Specially, it could act as the nanosized cross‐linking agent that reacts with thiolated HA via Michael addition. The biocompatibility, gelling ability, tissue adhesion, free radical scavenging, and bacterial inhibition of the hybrid hydrogel were evaluated. The potential bacterial inhibition and good tissue adhesion endow the hydrogel with great potential for wound dressing.

**Scheme 1:**
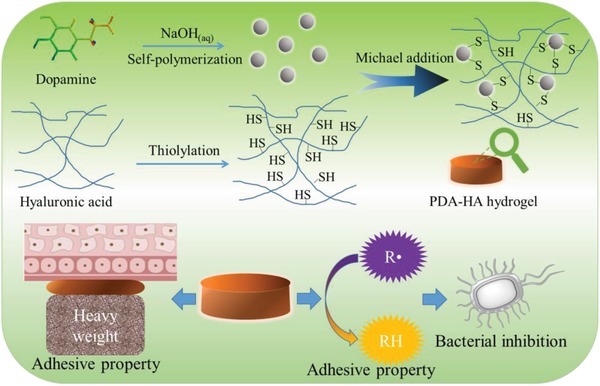
Schematic illustration of preparing the multifunctional PDA‐HA hydrogel with tissue adhesion, free radical scavenging, and bacterial inhibition.

As shown in **Figure** 1A, thiolated hyaluronic acid (HA‐SH) was obtained by two steps that referred to the amidation of HA with cystamine dihydrochloride (CD) and disulfide bond cleavage. The successful amidation of HA was verified by Fourier transform infrared (FT‐IR) and ^1^H NMR. Comparing with the FT‐IR spectrum of pure HA that exhibited the characteristic absorption at 1624 cm^−1^ for asymmetric carbonyl stretching vibration, the FT‐IR spectrum of HA‐SH exhibited two peaks at 1654 and 1553 cm^−1^ (Figure S1, Supporting Information), which are attributed to the amide I bond and amide II bond.^19^ The appearance of amide absorption reveals the successful amidation. Then, ^1^H NMR was introduced to investigate the grafted rate of HA‐CD. The ^1^H NMR spectrum of pure HA in D_2_O exhibited signals at 1.95 and 3.28–4.75 ppm (Figure 1B), which are attributed to the protons of CH_3_CO‐ and the sugar ring of hyaluronic acid unit, respectively. After the grafting of CD to carboxyl group by amidation reaction, new peaks from 2.68 to 2.94 ppm could be detected. These peaks are assigned to the chemical shift of ethyl protons in CD group. The appearance of these peaks also indicates the successful amidation reaction. In the presence of dithiothreitol (DTT), the disulfide bond was cleaved and yielded HA‐SH, accompanying with the shift of chemical shift from 2.68–2.94 to 2.45 ppm. The grafting degree of sulfhydryl group was 38%, which was determined by the protons of CH_3_CO‐ group of HA and the protons of HS‐CH_2_CH_2_NH‐. Specially, the thiolated graft rate could be controlled by adjusting the amount of activating agents and reaction time. As shown in **Table** 1, the thiolated graft rate is proportional to the activating agent content and reaction time. In consideration of these results, HA (4–10 kDa) with thiolated graft rate of 38% was used in the stepwise experiments.

**Figure 1:**
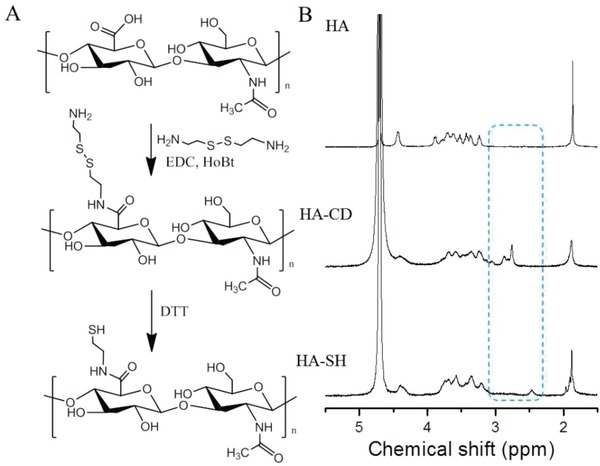
A) Schematic representation of synthetic route of HA‐SH. B) The corresponding ^1^H NMR spectra of HA, HA‐CD, and HA‐SH, respectively. The solvent is D_2_O.

**Table 1:** The influence of feed ratio and react time on the thiolated graft rate

HA *M*_w_ [kDa]	Feed ratio [g/g] EDC/HOBt/CD	React time [h]	Thiolated graft rate (%)
4–10	2/1.5/2	12	38
	2/1.5/2	24	78
	4/3/2	24	90
200–400	2/1.5/2	12	37.5
	2/1.5/2	24	50
	4/3/2	24	66

An alkali‐induced dopamine polymerization was achieved in NaOH solution and the morphology of PDA was imaged by scanning electron microscopy (SEM). According to **Figure** 2A, PDA exhibits a spherical structure with an average diameter of ≈400 nm. The diameter of PDA nanoparticles was further measured by dynamic light scattering (DLS), showing the average size 398 nm (Figure S2, Supporting Information). The average size of obtained PDA nanoparticles was larger than our previous report that the average size of PDA was 270 nm.^20^ The discrepancy between the PDA sizes should be ascribed to the different polymerization conditions.

**Figure 2:**
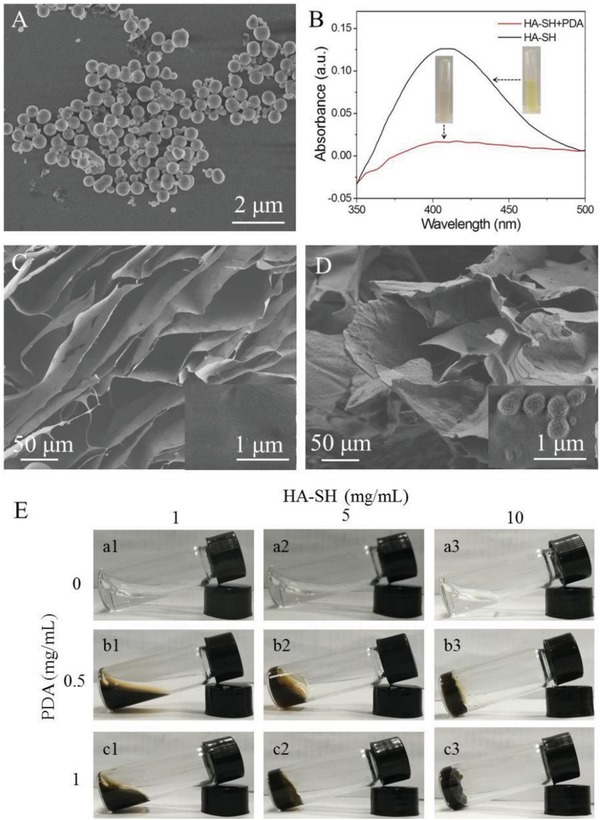
A) SEM image of PDA nanoparticles. B) The UV–vis spectra of HA‐SH and PDA‐HA. C,D) SEM images of pure HA‐SH and PDA‐HA hydrogel. The surface morphologies were inserted accordingly. E) Photos of HA‐SH and PDA‐HA hydrosol/hydrogel obtained from different HA‐SH and PDA concentrations.

To construct the PDA‐HA hydrogel, PDA dispersion was added to HA‐SH solution to initiate the Michael addition reaction between the catechol group of PDA chains and the thiol group of HA‐SH. The reaction process between PDA and HA‐SH was confirmed by Ellman's method. After adding PDA into the HA‐SH solution, as shown in Figure 2B, the solution underwent a color change from original yellow to colorless. In the meanwhile, the absorbance at ≈410 nm in the UV–vis spectrum exhibited a substantial decrease, suggesting the decrease of thiol group content. The result indicated that PDA could act as a nanosized cross‐linking agent to cross‐link HA‐SH. Certainly, reversible noncovalent interactions including π–π stacking from PDA and hydrogen bond from HA also contributed to the formation of hybrid PDA‐HA hydrogel.

SEM image showed that the pure HA hydrogel displayed porous structure (Figure 2C). After magnifying the image, we found that the pure HA hydrogel had a smooth surface (Figure 2C, inserted). In contrast to pure HA hydrogel, the PDA‐HA hydrogel exhibited less pores (Figure 2D). Specially, the surface of HA hydrogel was less smooth than that of the pure HA hydrogel. The magnified image showed the presence of PDA nanoparticles on the HA surface (Figure 2D, inserted), which further led to the interweaved 3D structure in the PDA‐HA hydrogel.

To investigate the influence of PDA concentration and molecular weight on the gelation ability of PDA‐HA, HA with molecular weights of 4–10 kDa and 20–40 wDa was investigated and different amounts of PDA were added. As shown in **Table** 2, increasing the concentration or molecular weight of pure HA‐SH could result in the gelation, arising from the intermolecular hydrogen bond interaction. Notably, the gelation time could be substantially shorted after the adding of PDA due to the cross‐linking reaction. The PDA concentration also influenced the gelation ability of HA‐SH. As shown in Figure 2E, pure HA with molecular weights of 20–40 wDa could not form the hydrogel at the observed concentration ranging from 1 to 10 mg mL^−1^. After the adding of PDA with 0.5 mg mL^−1^, the viscosity of HA increased and gelation occurred at 10 mg mL^−1^. When the PDA concentration was increased to 1 mg mL^−1^, HA could form hydrogel at 5 mg mL^−1^. These results indicated that the PDA doping would decrease the critical gel concentration of HA. Meanwhile, the size of PDA also has an effect on the performance of the hydrogel. PDA with small size was suboptimal for the formation of hybrid hydrogel, whereas large‐sized PDA exhibited undesirable dispersion in aqueous solution and might result in heterogeneous HA hydrogel. Thus, PDA with average size of ≈400 nm was introduced to cross‐link HS‐HA.

**Table 2:** Gel time of pure HA‐SH and PDA‐HA (PDA concentration is 1 mg mL^−1^)

	*C*_HA‐SH_ [mg mL^−1^]	HA‐SH gelation time	HA‐SH+PDA gelation time
4–10 kDa (degree of substitution (DS) = 38%)	1	nongelation	nongelation
	10	nongelation	>24 h
	20	≈24 h	≈12 min
20–40 wDa (DS = 37.5%)	1	nongelation	nongelation
	10	≈48 h	≈5 min
	20	≈1 h	≈5 min

The reaction conditions such as the polymerization time, alkaline and oxygen content would affect the self‐polymerization of dopamine, which further influence the addition reaction between HA‐SH and PDA. Through controlling the polymerization time of dopamine, size‐adjustable PDA‐HA hydrogel could be obtained. After adding the PDA nanoparticles that were obtained by self‐polymerization for 24 h into the HA‐SH solution, the PDA‐HA hydrogel formed due to the cross‐linking reaction mentioned above. Interestingly, the hydrogel begun to shrink after a while (Figure S3, Supporting Information). The size‐shrinkage of PDA‐HA hydrogel might be attributed to the oxidation cross‐linking among the residual thiol groups in the presence of a small amount of oxidant. To verify the hypothesis, DTT was added, which led to the effective expansion of cross‐linked PDA‐HA hydrogel. The DTT‐induced disulfide bond cleavage indicated the existence of oxidation cross‐linking. The size‐adjustable property of PDA‐HA hydrogel shows the great potential for the controllable drug release.

Cytotoxicity is the first concern of hydrogels for their biomedical applications. The HEK‐a cell was exploited to evaluate the biocompatibility of HA‐SH and HA‐PDA hydrogels. Epidermic cell line was introduced as the cell model due to the potential application in wound dressings. As shown in **Figure** 3A, HA‐SH exhibits no cytotoxicity against epidermic cells in our observed concentration range. After the cross‐linking with PDA, the obtained HA‐PDA not only shows good biocompatibility to mammalian cells, but also could promote the cell growth to some degree (Figure 3B). Along with the concentration increasing, the cell viability increased gradually compared with the control group. The promoted cell growth was attributed to the strong radical scavenging property of PDA, which could protect the cells against oxidative stress.^21^

**Figure 3:**
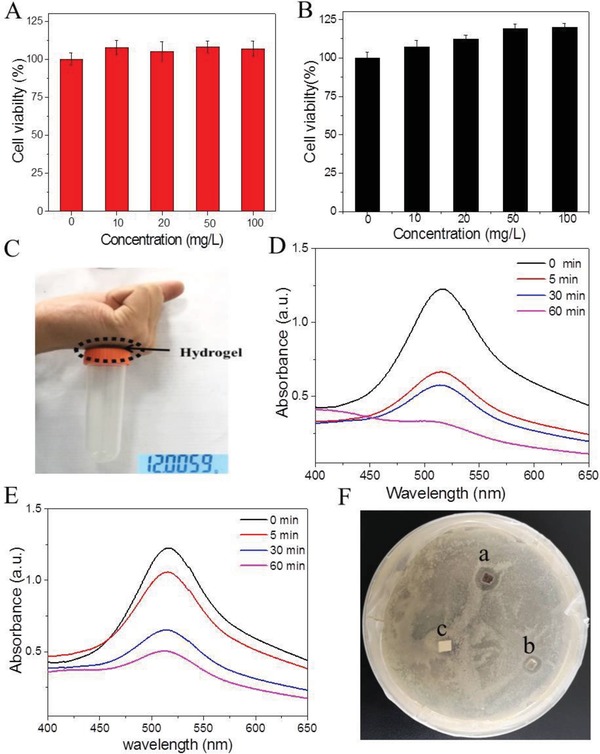
Cell viability profiles of HEK‐a cells after the coincubation with A) HA‐SH and B) PDA‐HA for 72 h, respectively. C) Tissue adhesiveness of the PDA‐HA hydrogel. The UV–vis spectra of DPPH after the coincubation with D) PDA‐HA and E) HA‐SH. F) The surface antibacterial activity test of PDA‐HA, HA‐SH hydrogels against *E. coli*, and filter paper was used as control; a) the PDA‐HA hydrogel, b) the HA‐SH hydrogel, and c) the filter paper as the blank.

If the hydrogels used for tissue repair and wound dressing equipped with excellent cell affinity, they can accelerate wound healing and tissue regeneration after implantation.^6^, ^22^ Due to the structural similarity with the adhesive protein of mussels, PDA exhibits excellent cell affinity.^23^ Therefore, PDA‐bearing HA hydrogel may also have good cell affinity and tissue adhesiveness. As shown in Figure 3C, the hydrogel could not only tightly adhere on the author's hand, but also hang some objects due to the robust adhesion. The PDA‐HA hydrogel formed by 20 mg mL^−1^ HA‐SH and 2 mg mL^−1^ PDA can support 12.2 g object. The good adhesion of HA‐PDA hydrogel was attributed to the introduction of PDA, since the pure HA hydrogel could not adhere any objects. Then, the influence of PDA concentration on the adhesion of HA‐PDA was investigated, accordingly. The weight of adhesive object was proportional to the PDA content in the hybrid HA‐PDA hydrogel (Table S1, Supporting Information).

As for wound dressings, inhibiting the inflammation is the desirable requirement. The occurrence of inflammation is generally associated with the generation of free radical species. It has been well documented that the PDA nanoparticles with phenolic groups can scavenge free radicals.^24^, ^25^ Herein, 2,2‐Diphenyl‐1‐picrylhydrazyl (DPPH) as the stable radical was used to investigate the radical scavenging activity of HA‐PDA hydrogel. As shown in Figure S4A in the Supporting Information, the pure DPPH solution showed the purple color, which faded into light orange when incubated with HA‐PDA hydrogel. The color change indicated the rapid disappearance of DPPH free radical. Meanwhile, the free radical scavenging activity of HA‐PDA hydrogel was conducted quantitatively by monitoring the absorbance at 516 nm at different time intervals. The absorbance at 516 nm showed a time‐dependent decrease and 73.8% of free radicals were scavenged by HA‐PDA hydrogel after 60 min (Figure 3D). The effective free radical scavenging might be attributed to the synergy between HA‐SH and PDA. To verify this inference, the free radical scavenging of free HA‐SH was also investigated, accordingly. According to Figure 3E, free HA‐SH indeed could capture the free radical effectively. 58.7% of free radicals could be scavenged by free HA‐SH hydrogel after 60 min incubation, and the original color also became weaker. The free radical scavenging of free HA‐SH was related with the presence of thiol group in HA‐SH.

Bacterial infection would generally increase exudate formation and delay wound healing process. Wound dressing containing antimicrobial agent can promote the wound healing process by reducing the number of pathogens and the inflammatory response of the wound site. Thus, an ideal wound dressing should possess antimicrobial property against the infection. The antibacterial activity of PDA‐HA hydrogel against *Escherichia coli* (Gram‐negative bacterium) was evaluated using surface antibacterial activity test. The size of the inhibition zone shows the antibacterial ability of the hydrogels. According to Figure 3F, the inhibition zone of PDA‐HA hydrogel (marked by a) is bigger than that of HA‐SH hydrogel (marked by b). In the control group, filter paper as the blank shows no inhibition zone (marked by c). The inhibition zone assay suggested both HA‐SH and PDA‐HA hydrogel owned the antibacterial ability, but the introduction of PDA into HA hydrogel could augment the antibacterial ability of hydrogel. These results were coincided well with the free radical scavenging test.

In summary, a novel hybrid PDA‐HA hydrogel was successfully fabricated via Michael addition reaction between PDA and HA‐SH. The introduction of PDA into HA‐SH conferred the hybrid hydrogel with various merits such as low critical gel concentration, improved the cell affinity and tissue adhesion, and efficient free radical scavenging ability. Specially, this PDA‐bearing hybrid hydrogel can efficiently inhibit bacterial growth, which will extend the possible applications of this promising material as wound dressings.

## Experimental Section

*Materials*: Hyaluronic acid (HA, *M*_W_ 4–10 kDa and 20–40 wDa) was purchased from Freda Biochem Co., Ltd. (Shandong, China). Dopamine hydrochloride was obtained from Aladdin. 1‐Ethyl‐3(3‐dimethylaminopropyl) carbodiimide (EDC), DTT, cystamine dihydrochloride (CD), DPPH and the Ellman reagent (5,5′‐dithiobis‐2‐nitrobenzoic acid, DTNB) were received from Shanghai yuanye Bio‐Technology Co. Ltd (China). *N*‐hydroxybenzotriazole (HOBt) was purchased from GL Biochem (Shanghai) Ltd. (China). Dopamine was obtained from Aladdin. Dulbecco's modification of Eagle's medium Dulbecco (DMEM), fetal bovine serum, penicillin and streptomycin were purchased from Gibco Life Technologies. Cell Counting Kit‐8 (CCK‐8) was provided by Dojindo China CO., Ltd. HEK‐a cells were obtained from BeNa culture collection.

*Instruments and Methods*: Proton nuclear resonance spectroscopy (^1^H NMR) was carried out on a Varian Mercury‐VX 300 spectrometer using D_2_O as solvent. The morphology of samples was imaged on a Nova NanoSEM FEI (Holland) instrument. The average size of PDA nanoparticles was recorded by Nano‐ZS ZEN3600 apparatus (Malvern Instruments) at room temperature. FT‐IR spectra were recorded on an AVATAR 360 spectrometer. The UV–vis absorbance of samples was recorded using a spectrophotometer (UV‐2550; Shimadzu, Kyoto, Japan).

*Synthesis and Characterization of Thiolated Hyaluronic Acid (HA‐SH)*: Thiolation of hyaluronic acid was accomplished by a facile two‐step synthetic method that involved the amidation of hyaluronic acid with cystamine dihydrochloride and subsequent disulfide bond cleavage. Firstly, 0.5 g of HA was dissolved with 30 mL of deionized water and swelled for 12 h. Then, 2 g of EDC, 1.5 g of HOBT, and 2 g of cystamine dihydrochloride were added into this system, and the pH value was adjusted to 6.8. After stirring for 12 h at room temperature, the cocktail was poured into dialysis membrane with molecular weight cutoff of 3500, which was further subjected to dialysis against deionized water for two days to remove the residues. Next, 1 g of DTT was added to cleave the disulfide bond at pH 8.5. After that, 0.5% (w/v) NaCl was added and the pH value was further adjusted to 3.5. The obtained HA‐SH was precipitated with ethanol and the precipitation was centrifuged and washed three times. Finally, the obtained HA‐SH was dried under vacuum in 40 °C for 12 h. The ^1^H NMR spectra were recorded using D_2_O as the solvent. The thiolated graft rate was calculated according to the following formula: $\eta =\left(\frac{A_{b}}{A_{a}}÷\frac{A_{b_{0}}}{A_{a_{0}}}\right)\times 100$, where *A*_b_/*A*_a_ is the observed peak area ratio of protons between HS‐CH_2_CH_2_‐ and ‐COCH_3_ from NMR spectrum, and *A*_bo_/*A*_ao_ denotes the theoretical peak area ratio (*A*_bo_/*A*_ao_ = 2/3).

*Synthesis of Polydopamine*: 4 mg mL^−1^ of dopamine hydrochloride was dissolved in 0.01 m NaOH and stirred for 24 h for the auto‐polymerization of dopamine. Then, the mixture was centrifuged at 12 000 rpm and the black precipitation was collected and washed three times with deionized water and two times with ethanol. Polydopamine was dried under vacuum in 40 °C for 12 h and stored at 4 °C.

*Preparation of Hydrogel and Its Adhesion Testing*: The PDA‐HA hydrogel was prepared by the following procedures. A certain amount of HA‐SH was dissolved with water and 0.02 m NaOH was added to assist the dissolvation of HA in a vial. A certain amount of PDA powder was dispersed with DMSO and added to the HA‐SH solution under ultrasonic treatment. As the control group, pure HA‐SH with same concentration was assistantly dissolved with 0.02 m NaOH.

The adhesion property of PDA‐HA hydrogel was evaluated by object suspension method, which involved that the hydrogel was sticked to the author's hand and the other side of the hydrogel hung the centrifuge tube. The weight of heavy object was the criteria to judge the adhesion property. A piece of cylindrical hydrogel with a diameter of 1 cm and height of 0.25 cm was taken out from vial and used for the adhesion evaluation.

*Ellman's Reaction*: The amount of thiol content in HA‐SH was assayed to investigate the gelling process of PDA‐HA using an Ellman's reagent.^26^ Briefly, 1 mg mL^−1^ HA‐SH and 0.01 mg mL^−1^ PDA were separately prepared and dispersed with 0.02 m NaOH solution. 4 mg of 5,5′‐dithiobis‐2‐nitrobenzoic acid (DNTB) were dissolved with 100 mL of NaOH (0.02 m). After that, 5 mL of 0.04 mg mL^−1^ DNTB solution was mixed with the cocktail for 1 min. The color change of the mixture was imaged and the UV–vis spectrum of the mixture was recorded from 350 to 500 nm. For the control group, 1 mg mL^−1^ HA‐SH was prepared with 0.02 m NaOH and added with DNTB solution for assay.

*Free Radical Scavenging Ability*: HA‐SH hydrogel and PDA‐HA hydrogel were prepared in the vials according to the above procedure. The HA‐SH concentration of both hydrogels was 20 mg mL^−1^ and the PDA concentration in PDA‐HA hydrogel was 1 mg mL^−1^. 4 mg of DPPH was dissolved in 2 mL of ethanol and 500 µL of DPPH solution was added to the hydrogels. At different time intervals (0, 5, 30, and 60 min), 30 µL of upper solution was taken out and diluted with 1 mL of ethanol. The color of the solution would be monitored and UV–vis spectra were recorded to calculate free radical scavenging rate. The relative free radical scavenging rate was calculated according to the formula:

DPPH scavenging (%) = (*A*_B_ − *A*_S_)/*A*_B_ × 100, where A_S_ stands for the absorbance of the sample after mixing with DPPH, and *A*_B_ represents the absorbance of DPPH in ethanol with the same concentration of the experimental group.

*In vitro Cytotoxicity Assay*: HEK‐a cells seeded in a 96‐well plate were cultured in 100 µL of DMEM containing 10% FBS for one night under a humidified 5% CO_2_ atm. Subsequently, 20 µL of HA‐SH or PDA‐HA dispersed in 180 µL of DMEM were added to each well, and the cells were incubated for another 72 h in the dark. Then, the medium was replaced with 100 µL of fresh CCK8 (CCK:DMEM = 1:10). After 1 h of incubation, the optical density (OD) was measured at 450 nm with a microplate reader model. The average value of the experiments was collected, and the cell viability could be obtained as follows:

Cell viability (%) = (OD_sample_/OD_control_) × 100, where OD_control_ is obtained in the absence of HA‐SH or PDA‐HA, and OD_sample_ is obtained in the presence of HA‐SH or PDA‐HA.

*Antibacterial Ability*: E. coil was incubated in 50 mL of beef extract peptone medium at 37 °C for 24 h. Then the medium was poured into a petri dish in a sterile environment. After the solidification, 100 µL of E. coil was coated on the plate. The HA‐SH and PDA‐HA hydrogels with same size were applied to the plate along with a sterilized filter paper and cultured at 37 °C for another 24 h. The concentration of HA‐SH for both groups was 20 mg mL^−1^. And the concentration of PDA was 2 mg mL^−1^.

## Conflict of Interest

The authors declare no conflict of interest.

## Supporting information

Supporting Information
