# Supplementary material for: Mussel‐Inspired Adhesive Polydopamine‐Functionalized Hyaluronic Acid Hydrogel with Potential Bacterial Inhibition
Source: Glob Chall. 2019 Nov 18;4(2):1900068. doi: 10.1002/gch2.201900068 (PMC7001117; doi:10.1002/gch2.201900068)
Supplement: Supplementary file 1 — Supporting Information [file GCH2-4-1900068-s001.pdf]

Copyright WILEY-VCH Verlag GmbH & Co. KGaA, 69469 Weinheim, Germany, 2019.

Global Challenges
Open Access

Supporting Information

for *Global Challenges,* DOI: 10.1002/gch2.201900068

Mussel-Inspired Adhesive Polydopamine-Functionalized Hyaluronic Acid Hydrogel with Potential Bacterial Inhibition

Qi-Hang Yu, Chen-Ming Zhang, Zhi-Wei Jiang, Si-Yong Qin, and Ai-Qing Zhang*

Copyright WILEY-VCH Verlag GmbH & Co. KGaA, 69469 Weinheim, Germany, 2019.

Supporting Information

Mussel-inspired Adhesive Polydopamine-functionalized Hyaluronic Acid Hydrogel with Potential Bacterial Inhibition

Qi-Hang Yu, Chen-Ming Zhang, Zhi-Wei Jiang, Si-Yong Qin, Ai-Qing Zhang*

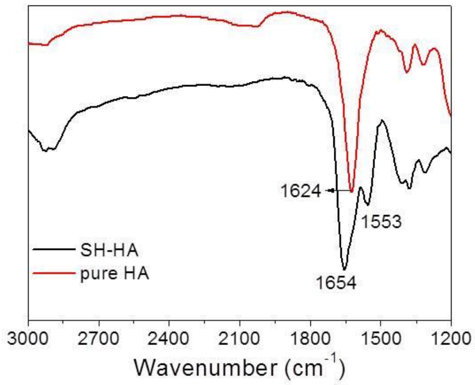

Figure S1. FT-IR Spectra of HA-SH and pure HA.

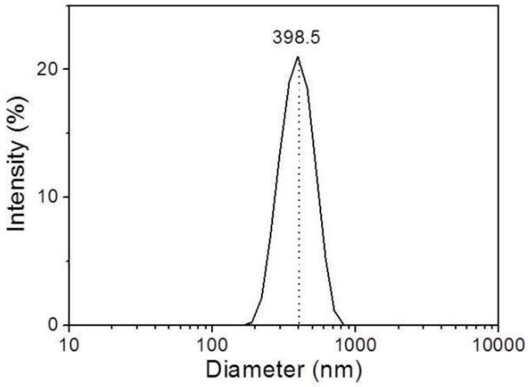

Figure S2. The size distribution of the PDA nanoparticles at room temperature.

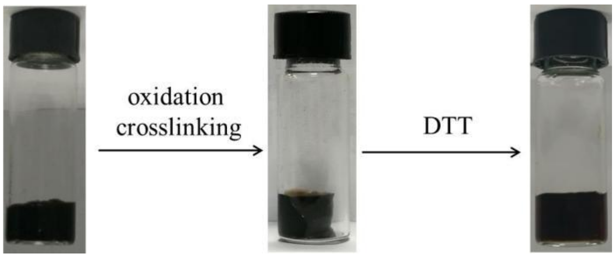

Figure S3. Photos of the size-adjustable PDA-HA hydrogel.

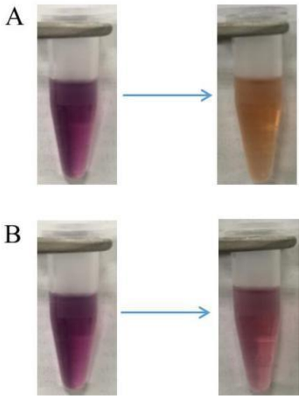

Figure S4. Photos of the color change of DPPH solution before and after the incubation of PDA-HA (A) and HA-SH (B).

Table S1. The adhesion of polydopamine-thiolated hyaluronic acid hydrogel (grafting rate=38%).

$C_{HA\text{-}SH}$	C_{PDA}	W_{object}
10 mg/ml	0 mg/mL	0 g
20 mg/ml	0 mg/mL	0 g
10 mg/ml	0.5 mg/mL	1.7 g
20 mg/ml	0.5 mg/mL	2.6 g
10 mg/ml	1 mg/mL	3.7 g
20 mg/ml	1 mg/mL	5.7 g
10 mg/ml	2 mg/mL	12.0 g
20 mg/ml	2 mg/mL	12.2 g
